# Investigating the impact of charge and hydrophilicity on peptide-mucin interactions using a simple mucin model

**DOI:** 10.1186/s41120-025-00123-5

**Published:** 2025-10-01

**Authors:** Waleed M. Elballa, Aiden Gregory, Teruna J. Siahaan, Michael J. Hageman

**Affiliations:** 1 Department of Pharmaceutical Chemistry, The University of Kansas, Simons Laboratories, Simons Biosciences Research Laboratories, 2093 Constant Ave, Lawrence, KS 66047, USA; 2 School of Pharmacy, The University of Kansas, 2010 Becker Dr, Lawrence, KS 66047, USA; 3 Present Address: Department of Pharmaceutical Sciences, Nesbitt School of Pharmacy, Wilkes University, 84 W South Street, Wilkes-Barre, PA 18766, USA

**Keywords:** Octreotide, Lanreotide, ADT peptides, Peptide-mucin interactions, Mono-compartment mucin model, Mucin diffusion, Cadherin peptides

## Abstract

*In vitro* models used to investigate drug diffusion face certain limitations and challenges because they omit for mucus interactions that could influence diffusional transport. This study developed a simple mono-component mucin model using Mucin Type II from porcine stomach to predict the effects of the physicochemical properties of peptides on their diffusion through the intestinal mucus layer. The diffusion of octreotide and lanreotide through a mucin layer was compared with their respective Ala mutants replacing Lys (i.e., octreotide A5 and lanreotide A5). Ala mutants showed higher diffusion than their respective parent peptides, implicating that the charge interaction between positively charged, Lys-containing peptide and negative charge mucin override their hydrophobic interactions, thus hindering peptide diffusion. This finding was also supported by the faster diffusion of the negatively charged FITC-ADT10 compared to the positively charged FITC-HAV10 peptide. Thus, the interaction between the peptide’s positive and the negative charge of the glycans in mucin hinders peptide diffusion.. The neutral DTPPVK has the highest hydrophilicity and diffusion compared to negatively charged DTPPD, DTPPT, and ADTC5. Although DTPPD and DTPPT have about the same hydrophilicity, DTPPD has better diffusion than DTPPT because DTPPD with −2 charges has higher negative charge repulsion against mucin compared to that of DTPPT with −1 charge. Finally, ADTC5, with the lowest hydrophilicity, has the lowest diffusion through the mucin layer. This study found that the charge and hydrophilicity of peptides influence their diffusion across the mucin layer, and these studies correlate with the previous studies utilizing different in vitro models.

## Introduction

The oral delivery of macromolecules (e.g., peptides, proteins, oligonucleotides) is hindered by the anatomy and physiology of the gastrointestinal tract (Subramanian et al. 2022). The intestinal epithelial cell layer and the apical mucus layer are among the major barriers to macromolecule absorption in the intestine (Karlsson et al. 1993). The mucus layer acts as the first barrier to drug oral absorption, and the physicochemical properties of the drugs, such as size, charge, and hydrophilicity, are all presumed to play an important role in their oral absorption. The mucin-drug interactions are due to electrostatic and hydrophobic interactions, as well as molecular size filtration (Pontremoli et al. 2015; Larhed et al. 1997; Caron et al. 2015). In addition, the mucus layer in the small intestine has a protective role in pathogenic infections because the small intestine is prone to a higher risk of infection in mammals (Syed et al. 2022). Conversely, this protective role reduces the diffusion of nutrients as well as both hydrophilic and hydrophobic drugs (Subramanian et al. 2022; Johansson and Hansson 2011; Meaney and O’Driscoll 1999).

Mucus mainly consists of 90–95% water with 5% mucin along with small quantities of lipids, proteins, DNA, and electrolytes (Boegh et al. 2015). Mucin is constructed from long-chain proteins that have domains composed of proline, threonine, and serine amino acids called “PTS” domains. These PTS domains are glycosylated with glycans attached to the side chains of the threonine and serine residues, each containing negatively charged sialic acid and sulfate groups; thus, PTS has a high overall negative charge (Subramanian et al. 2022; Dekker et al. 2002; Cone 2009). Mucin also has a cysteine-rich domain with disulfide bonds to cross-link mucin chains, resulting in stronger interchain interactions that enhance the overall crosslinking and physico-mechanical strength of mucin (Subramanian et al. 2022; Sheehan et al. 1986). Various studies reported the presence of different pore sizes as large as 211 nm in the mucin of the small intestine (Bajka et al. 2015). These pores can restrict the diffusion of 100 nm particles or molecules (Ensign et al. 2013; Abdulkarim et al. 2015). Particle diffusion is also affected by intermolecular interactions between particles or molecules and the mucus layer (Subramanian et al. 2022; Yildiz et al. 2015). In this case, molecules with molecular weights larger than 1.0 kDa can form monovalent and polyvalent electrostatic interactions with mucin fibers, which hinder their diffusion through the mucin network (Subramanian et al. 2022; Larhed et al. 1997). For example, chitosan and other cationic molecules can form polyvalent bonds with the negatively charged glycan, resulting in mucoadhesion properties (Subramanian et al. 2022; Svensson and Arnebrant 2010). Oil-loving compounds diffuse more slowly, but do diffuse, through the mucus layer than water, rationalized by the presence of hydrophobic and cysteine-rich domains within the mucin structure (Subramanian et al. 2022; Matthes et al. 1992).

The drug molecules must pass through the mucin layer to be absorbed and transferred across the intestine to enter the circulatory system. The drug-mucus interactions could impede the permeation of drugs across the intestinal epithelial cell layer into the systemic circulation to reach the target tissue to elicit their biological response. Therefore, a high drug binding to mucin may reduce its diffusion rate as well as its overall exposure and effectiveness (Pontremoli et al. 2015). For example, Karlsson et al. found that the mucus layer was the largest barrier to the absorption of testosterone because it can interact with both extracellular and intercellular mucin molecules (Karlsson et al. 1993). Thus, lipophilic drugs like testosterone have affinity for the non-glycosylated region of mucin, hindering their diffusion through the mucus layer compared to more hydrophilic drugs. Similarly, positively charged drugs electrostatically bind to the negatively charged glycans of mucin, impeding diffusion through the mucus layer (Falavigna et al. 2020; Sigurdsson et al. 2013). The increase in mucin concentrations decreased the diffusion rates of atenolol, caffeine, and naproxen; however, the extent of the decrease in diffusivity varied depending on their interactions with the mucin layer (Falavigna et al. 2020). As a hydrophobic cyclic peptide, cyclosporin A (CsA) interacts with intestinal (MUC2), gastric (MUC5AC), and salivary (MUC5B) mucus to form an aggregation of polymeric and gel-forming mucins in its presence (Kishimoto et al. 2022). The magnitude of drug binding affinity to mucin does not correlate well with the drug’s physicochemical properties; therefore, other factors could be involved in drug permeation through the mucin layer (Kishimoto et al. 2024). Therefore, the ability to quickly and simply evaluate the potential for impacts on absorption is critical.

Ala-Asp-Thr (ADT) and His-Ala-Val (HAV) peptides were derived from the EC1 domain of E-cadherin protein. These peptides can modulate the cadherin-cadherin interaction in the *adheren* junctions of biological barriers to increase the pore size of the intercellular junctions; therefore, they can improve the apparent permeability of large hydrophilic molecules across the biological barriers (Kiptoo et al. 2011). Cyclic ADT peptides increased porosity of the paracellular pathway of Madin-Darby Canine Kidney cell monolayers (MDCK); MDCK monolayer has been widely used as a model to investigate drug transport across the biological barriers (Laksitorini et al. 2015). ADTC5 peptide (Table 1) has improved the delivery of hydrophilic marker molecules, such as ^14^C-Mannitol and Gd-DTPA across the blood–brain barrier in vitro and in vivo (Laksitorini et al. 2015). ADTC5 peptide improved the delivery of IgG monoclonal antibodies (mAb) to the brains of C57BL/6 mice by modulating the intercellular junctions in the blood–brain barrier (BBB) (Ulapane et al. 2019). Additionally, ADTC5 and HAVN1 cyclic peptides have improved the oral bioavailability of 4000 Da fluorescein-isothiocyanate dextran (FD4) by 4.4 and 7.2-fold, respectively, following intrajejunal administration (Dening et al. 2021). This finding indicates that ADT and HAV peptides can also modulate the intercellular junction in the intestinal mucosa. During this study, it was also noticed that HAVN1 peptide exhibited a delayed T_max,_ but not for ADTC5 peptide (Dening et al. 2021). The hypothesis was that HAVN1 peptide could have interacted with the mucus barrier due to the presence of a basic His residue within the peptide, which can undergo electrostatic interaction with the negatively charged mucin in the mucus layer. On the other hand, ADTC5 contains the negatively charged Asp, which may facilitate its diffusion across the mucus barrier (Dening et al. 2021).

In vitro experimental models have been routinely used to study drug diffusion and interaction across the mucus layer; these models are based on native mucus collected from animals, simulated mucus, purified mucin, or cell culture models that tend to produce some surface mucin. Due to the difficulty in obtaining native mucus and the great variation of isolated mucus samples, it is still uncertain whether the isolated and stored mucus can fully represent the mucus properties in the in vivo system. The general limitation of the in vitro model is the absence of several physiological factors such as GI fluid, gastric motility, gastric emptying, blood flow, and lymph flow (Boegh et al. 2013). In vitro model results can even vary due to the cell passage number, culturing time, media, cell seeding density, and the type of semipermeable membrane used (Boegh et al. 2013).

In this study, we aimed to use a simple and straightforward model of mono-component purified mucin to differentiate the diffusion of linear and cyclic peptides through a mucus layer (Table 1). The effects of charge and hydrophobicity on the diffusion of octreotide and lanreotide and their Ala5 mutants (replacing charged Lys (K)) were evaluated. We also designed new derivatives of cyclic ADTC5 peptide (i.e., DTPPD, DTPPT, DTPPVK, Table 1) to vary their physicochemical properties (Table 1) to improve their mucus permeation. Therefore, the diffusive properties of these peptides were determined through a mucin layer to predict their ability to reach either epithelial cells of the intestinal mucosa barrier or the adherens of the tight junction.

## Materials and methods

### Material

Mucin type II from porcine stomach and Corning^®^ Transwell^®^ polyester membrane cell culture inserts (CLS3412 and CLS3460) were obtained from Millipore Sigma. Octreotide, octreotide A5, lanreotide, lanreotide A5, ADTC5, DTPPD, DTPPT, and DTPPV peptides (Table 1) were purchased from dgpeptide (Wuhan, China). FITC-ADT10, FITC-HAV10, and ADTC5 were synthesized using Fmoc chemistry in a solid-phase peptide synthesis method in an automated peptide synthesizer (Tribute, Gyros Technology, Tucson, AZ, USA) according to a previous study (Laksitorini et al. 2015).

### Preparation of mucin model

**Fig. 1:**
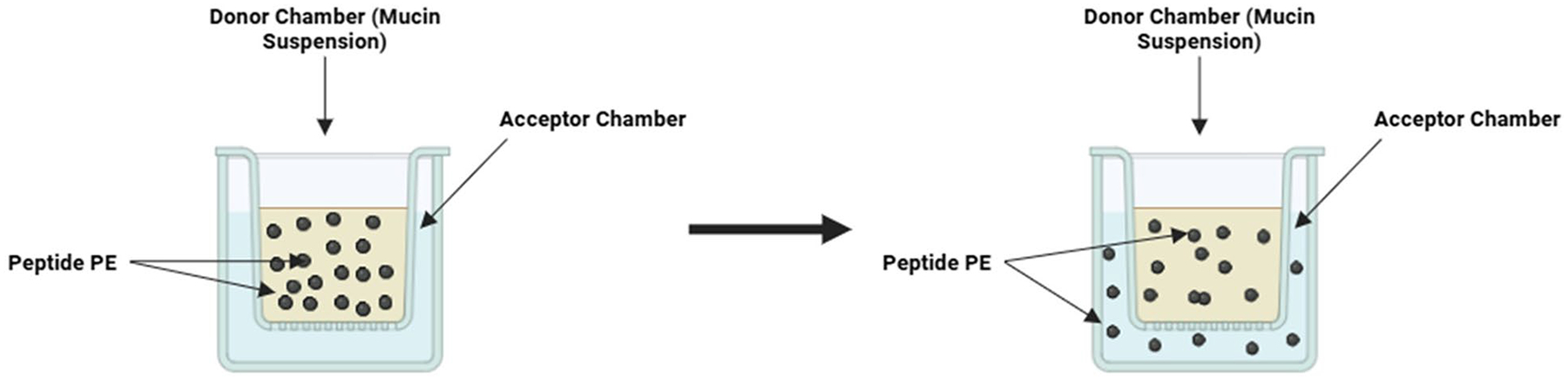
A schematic diagram of the diffusion chamber to study peptide diffusion through a mucin layer. The well consists of donor and acceptor chambers separated by a mucin layer. The peptide was added to the donor chamber, and the diffusion of the peptide was monitored by its appearance in the acceptor chamber. The peptide in the acceptor chamber was sampled as a function of time, and the peptide concentration was determined by fluorescence spectroscopy, HPLC, or mass spectrometry

Around 2.5% (w/v) of Type II porcine mucin was added to the donor side of the Transwell^™^ plate along with 300–1000 μL of 6.8 pH phosphate buffer. The mixture was shaken at 37°C for 45–60 min until the proper suspension was attained. Then, 1–2 mL of phosphate buffer at 6.8 pH was added to the acceptor side of the Transwell^™^ plate. A schematic diagram describing the model is shown in Fig. 1.

### Peptide diffusion across the mucin layer

Initially, FITC-ADT10 and FITC-HAV10 peptides were used to test the mucin model in phosphate buffer at pH 6.8. Different concentrations of peptide in phosphate buffer at pH 6.8 were added to the donor chamber; the acceptor chamber contained a phosphate buffer at pH 6.8 in the Transwell^™^. At predetermined time points, 100 μL aliquots were taken from the acceptor chamber, transferred into 96-well plates, and replaced with pre-heated phosphate buffer pH 6.8. The aliquots were then analyzed by fluorescence spectroscopy plate reader at λ_em_ = 495 nm and λ_ex_ = 519 nm.

The diffusion of each unlabeled peptide from the donor-to-acceptor chambers was analyzed by Agilent^®^ 1200 HPLC equipped with a Zorbax C18 column with 3.5 μm particle size and dimensions of 4.6 × 50 mm. The column temperature was held at 30°C during the chromatographic run. Mobile phase A was 0.1% trifluoroacetic acid in Milli-Q water, and mobile phase B was 0.085% trifluoroacetic acid in acetonitrile. The detector was a UV–Vis detector monitoring at 214 nm wavelengths. Calibration curves were prepared in phosphate buffer pH 6.8 in concentrations ranging from 1 to 266 μg/mL.

### Statistical analysis

Student t-tests were used to analyze the difference between the percentage diffused of FITC-HAV10 and FITC-ADT10, octreotide and octreotide A5, and lanreotide and lanreotide A5. The difference in diffusion percentages of ADT peptides was analyzed using ANOVA with Student − Newman − Keul post hoc comparison of the means and Dunnett’s Test comparing DTPPD, DTPPT, and DTPPVK with ADTC5. Statistical significance was set at p < 0.05 unless otherwise stated.

## Results

### FITC-HAV10 and FITC-ADT10

**Fig. 2:**
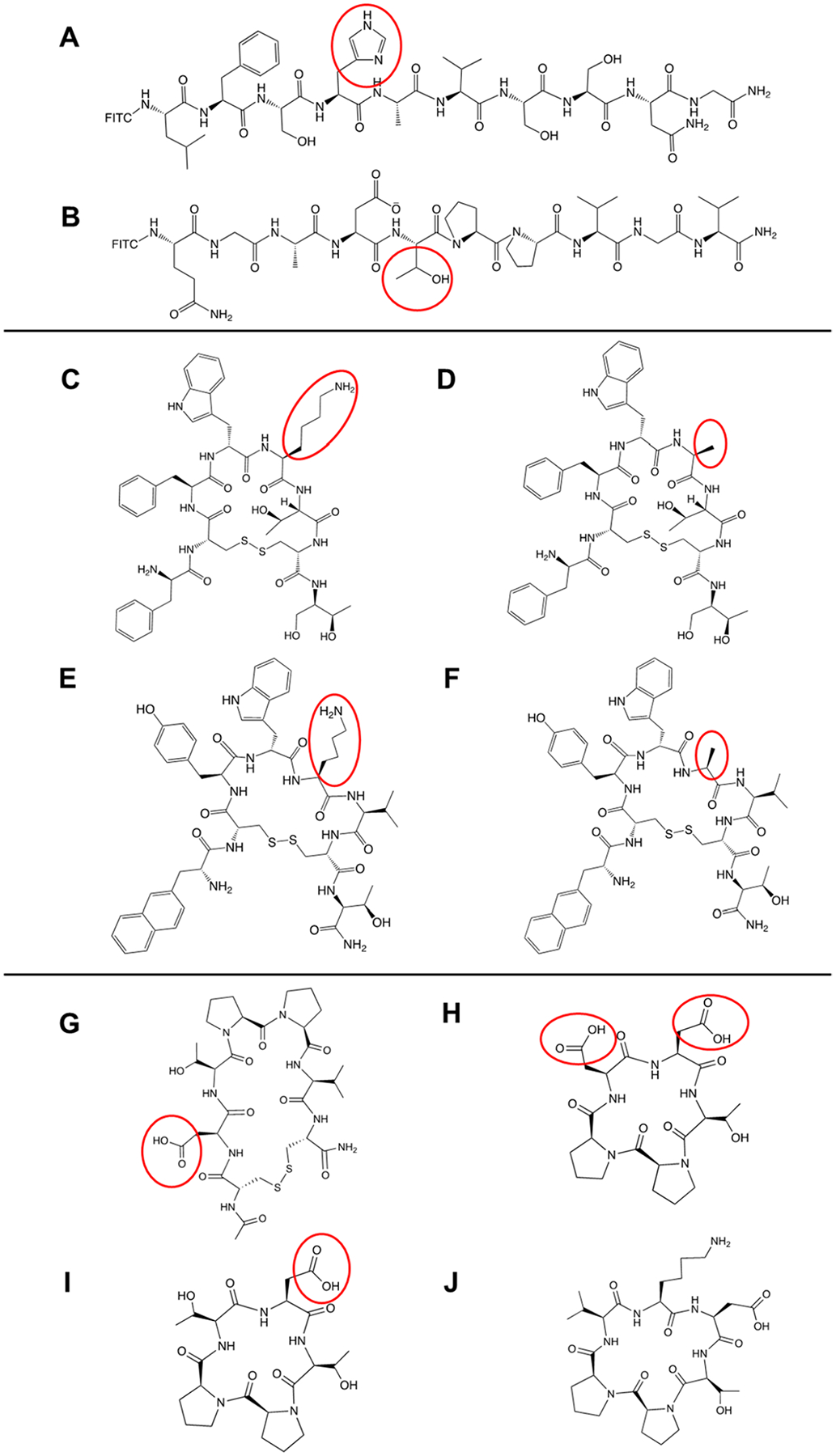
Chemical structures of peptides utilized in this study: **A** FITC-HAV10, **B** FITC-ADT10, **C** Octreotide, **D** Octreotide A5, **E** Lanreotide, **F** Lanreotide A5 **G** ADTC5, **H** DTPPD, **I** DTPPT, and **J** DTPPVK.Red circles highlight structural differences contributing to charge variation

**Fig. 3:**
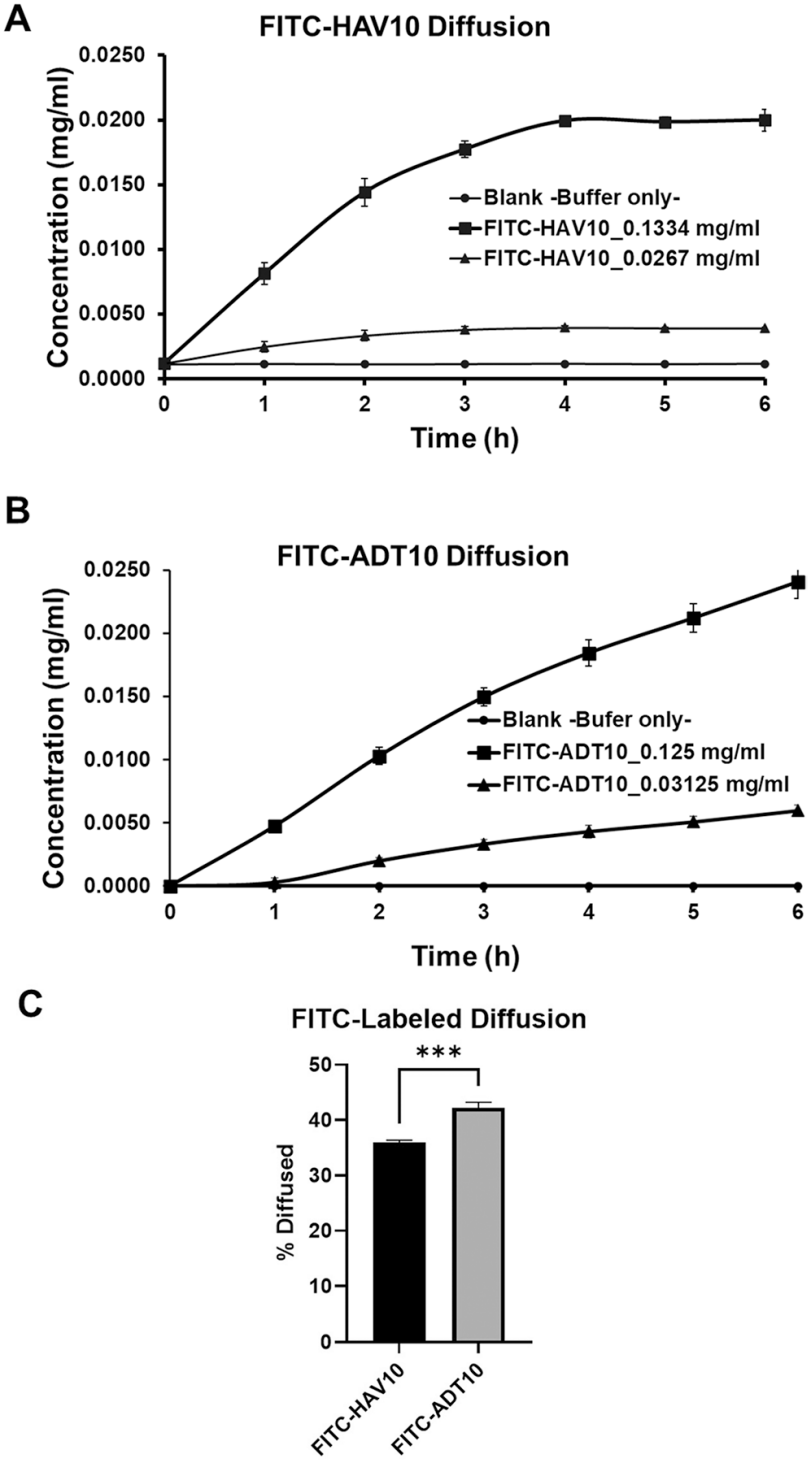
The concentrations of **A** FITC-HAV10 and **B** FITC-ADT10 peptides at the acceptor chamber after diffusing through the mucin as a function of time over 6 h. **C** Comparison of the average percentage of diffused FITC-HAV10 and FITC-ADT10 peptides -at 130 and 30 μg/ml concentrations- after 4 h

Initially, FITC-HAV10 and FITC-ADT10 peptides (Fig. 2A–B) were evaluated to test the mucin diffusion model; for the ease of analysis, samples were simply transferred to a 96-well plate and analyzed in a fluorescence plate reader. Flux experiments were carried out at a high (130 μg/mL) and a low concentration (30 μg/mL). The cumulative amounts of peptides over 6 h were detected in the acceptor chamber. At a high concentration, the accumulation of FITC-HAV10 in the acceptor chamber increased rapidly at 0–4 h and the concentration plateaued at 4–6 h with a maximum of 0.022 mg/mL (Fig. 3A). At low concentration, FITC-HAV10 peptide showed slower accumulation in the acceptor chamber compared to the high concentration (Fig. 3A). The slope increase at the low concentration was shallower and plateaued at 3–6 h compared to the high concentration.

The diffusion profile of FITC-ADT10 at a high concentration was different than that of FITC-HAV10. The accumulation of FITC-ADT10 was continuously increased at 0–6 h duration of time (Fig. 3B). At a low concentration, there was a lag time of peptide diffusion between 0-to-1 h time duration (Fig. 3B). This diffusion lag time was not observed with FITC-HAV10. To test whether the lag time is present in a high concentration (130 μg/mL), the diffusions of both peptides were monitored in the initial 30 min. Both FITC-HAV10 and FITC-ADT10 peptides showed a linear increase of concentrations at the acceptor chamber, suggesting that the lag time observed with FITC-ADT10 is likely due to sampling deviation (data not shown). Overall, the percentage of FITC-ADT10 diffused across the mucin layer was significantly higher than that of FITC-HAV10 at the 4 h time point (Fig. 3C).

### Diffusion of octreotide, octreotide A5, lanreotide, and lanreotide A5

The model was developed to evaluate the effects of the charges and hydrophobicity of cyclic peptides on their interactions with the mucin layer. The influence of these interactions on peptide diffusion across the mucin layer was evaluated using octreotide, octreotide A5, lanreotide, and lanreotide A5 peptides (Fig. 2A–F). At pH 7.0, octreotide and lanreotide have two positive charges from the N-terminus and the Lys5 residue side chain. The Lys5 residue in both octreotide and lanreotide was mutated to the Ala5 residue to produce octreotide A5 and lanreotide A5, respectively. Both octreotide A5 and lanreotide A5 have an overall charge of + 1 at pH 7. Two concentrations (266 and 133 μg/mL) from each peptide were used for their diffusion across the mucin layer. The concentrations of samples at the acceptor chamber were determined using analytical HPLC.

**Fig. 4:**
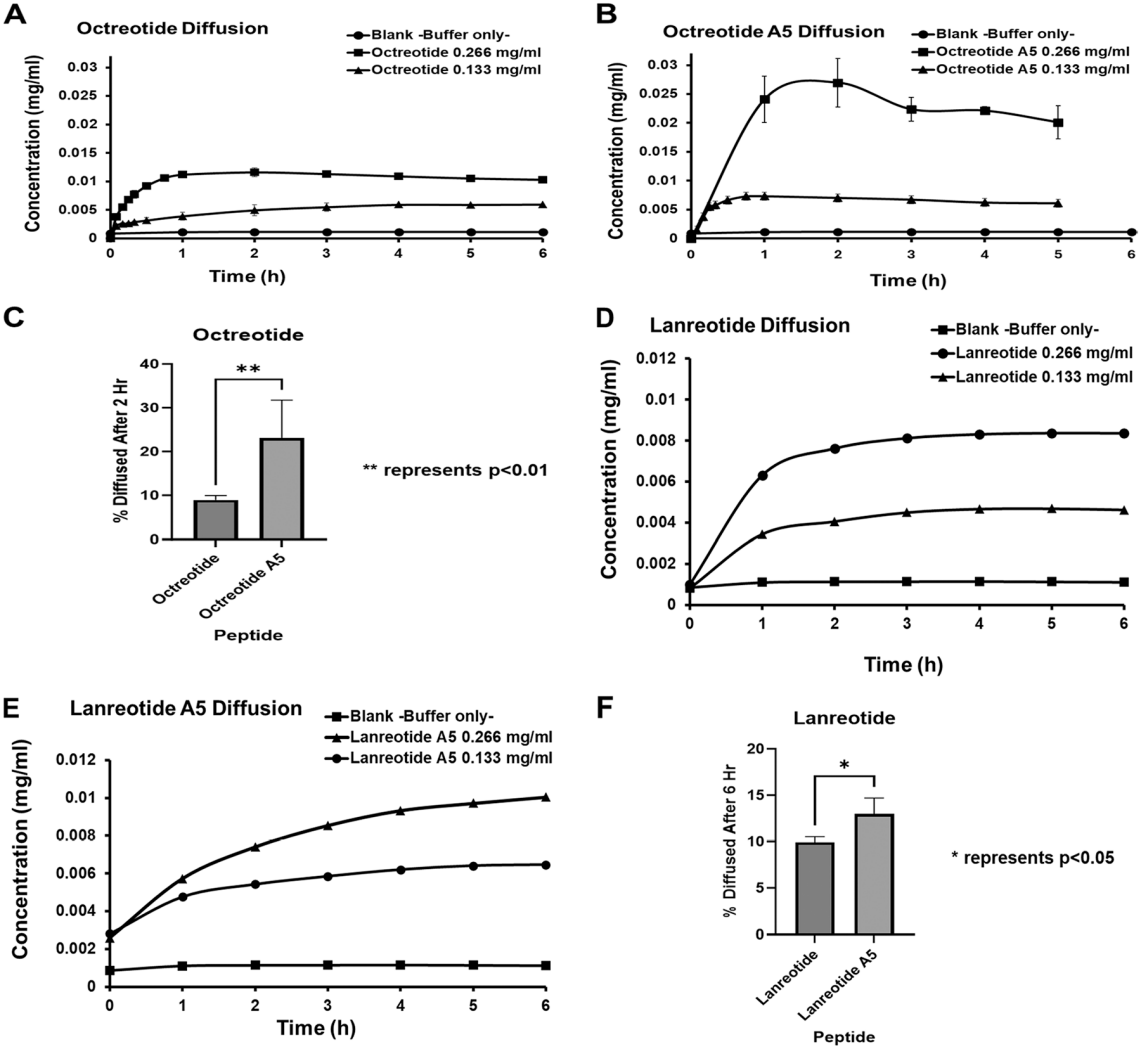
**A-B** Cumulative amounts of diffused peptides in the acceptor chamber of **A** Octreotide with + 2 charges and **B** Octreotide A5 with + 1 charge. **C** Comparison of the cumulative percentage of diffused Octreotide and Octreotide A5 up to the 2 h time point. **D-E** Cumulative amounts of diffused peptides in the acceptor chamber from **D** Lanreotide with + 2 charges and **E** Lanreotide A5 with + 1 charge. **F** Comparison of the cumulative percentage of diffused Lanreotide and Lanreotide A5 over a 2 h time point

At 266 μg/mL, octreotide accumulation in the acceptor chamber increased rapidly for the first 2 h; then, the concentration plateaued between 2–6 h with a maximum concentration of 0.0116 mg/mL (Fig. 4A). At 133 μg/mL, octreotide accumulation in the acceptor chamber increased rapidly at 0–4 h before plateauing between 4–6 h with a maximum concentration of 0.00593 mg/mL (Fig. 4A). On the other hand, octreotide A5 at 266 μg/mL accumulated in the acceptor chamber rapidly in the first 2 h with a plateau at 2–6 h and a maximum concentration of 0.0269 mg/mL (Fig. 4B). The maximum accumulation of octreotide A was twice that of octreotide. A similar diffusion profile was observed at 133 μg/mL for octreotide A5, where the accumulation increased rapidly in the first hour, followed by a plateau at 1–6 h (Fig. 4B). The maximum concentration of octreotide A5 was 0.0073 mg/mL, which was slightly higher than octreotide. The cumulative percentage diffused from octreotide A5 over 2 h was significantly higher than that of octreotide (Fig. 4C).

The diffusion profile of lanreotide at 266 and 133 μg/mL concentrations showed its accumulation in the acceptor chamber increased rapidly at 0–4 h, followed by a plateau at 4–6 h (Fig. 4D). In contrast, the diffusion profile of lanreotide A5 was different than that of lanreotide at both concentrations (266 and 133 μg/mL). Lanreotide A5 accumulation in the acceptor chamber increased rapidly at 0–4 h, and the accumulation kept increasing slowly at 4–6 h, unlike lanreotide (Fig. 4E). The cumulative percentage diffused from lanreotide A5 over 6 h was significantly higher than that of the parent lanreotide (Fig. 4F).

### Diffusion of cyclic ADT Peptides

The effects of charge and hydrophilicity were evaluated using ADTC5, DTPPVK, DTPPD, and DTPPT peptides. The peptide-mucin interactions influence their diffusion across the mucus layer. At pH 7.0, ADTC5 peptide bears one negative charge from the Asp2 residue (Fig. 2G). The DTPPD peptide has two negative charges from the Asp1 and Asp5 residues (Fig. 2H). The DTPPT peptide only has one negative charge from the Asp1 residue (Fig. 2I). Finally, the DTPPVK peptide has a neutral charge due to a charge cancellation between the Asp1 negative charge and the Lys6 positive charge (Fig. 2J). Two concentrations (266 and 133 μg/mL) from each peptide were used, followed by HPLC analysis of samples from the acceptor chamber.

**Fig. 5:**
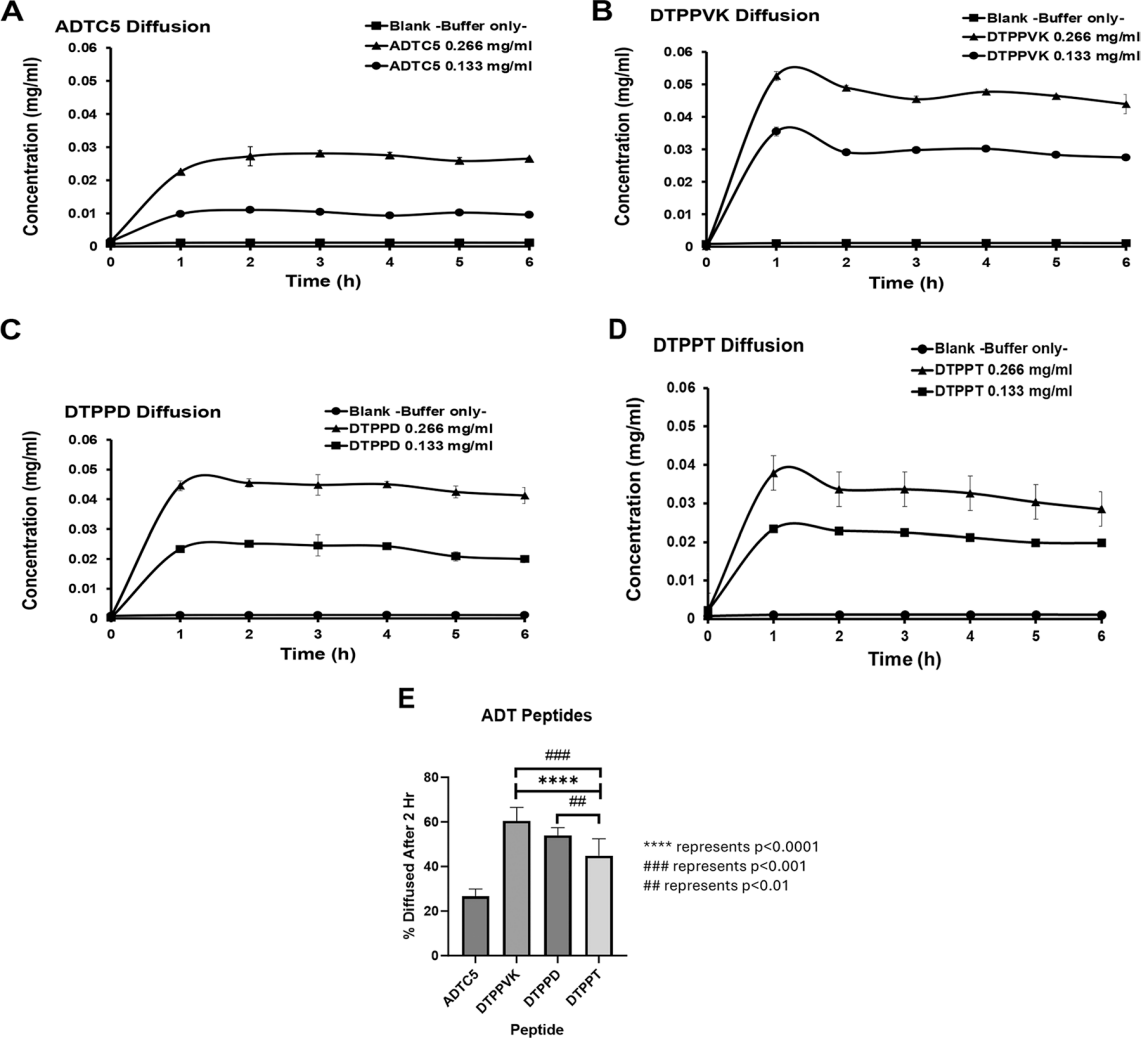
Cumulative amounts of diffused peptides in the acceptor chamber for **A** ADTC5, **B** DTPPVK, **C** DTPPD, and **D** DTPPT. Comparison of the cumulative percentages of diffused ADTC5, DTPPVK, DTPPD, and DTPPT peptides over 2 h

The diffusion profile of ADTC5 was similar at both 266 and 133 μg/mL, where the accumulation of ADTC5 in the acceptor chamber increased rapidly at 0–2 h, followed by a plateau at 2–6 h time points (Fig. 5A). The accumulation of DTPPVK at both concentrations increased rapidly in the first 1 h and leveled off at 1–6 h time points (Fig. 5B). The total accumulations of DTP-PVK in the acceptor chamber were higher than those of ADTC5 at 1–6 h. The diffusion of DTPPD showed a rapid increase in the acceptor chamber at 0–2 h along with a leveling off at 2–6 h duration (Fig. 5C). The maximum concentration of DTPPD in the acceptor chamber was also higher than ADTC5 but lower than DTPPVK. The diffusion profile of DTPPT peptide was similar to DTPPVK, and the accumulation of DTPPT increased rapidly in the first 1 h and leveled off at 1–6 h (Fig. 5D). The maximum accumulated concentration of DTPPT peptide was lower than that of DTPPVK peptide. Overall, the newly designed cyclic peptides (i.e., DTPPD, DTPPT, and DTPPVK) have significantly higher diffusions across the mucin layer compared to ADTC5 (Fig. 5E). Finally, the diffusion profiles of these peptides were in the following order: DTPPVK > DTPPD > DTPPT > ADTC5.

## Discussion

In the past, various models were used to assess the diffusion of molecules across the intestinal mucus layer, including the use of native mucus along with fluorescent recovery after photobleaching (FRAP) and multiple particle tracking (MPT) (Liu et al. 2021; Occhipinti and Griffiths 2008; Ernst et al. 2017). These methods are excellent for investigating the characteristics of molecule or particle transport at the microscopic level. However, the use of native mucus has limitations due to the availability and variation of mucus from different animal sources. The composition and property variation of mucus can generate inconsistency in the generated data. Several cell culture and co-culture models have been used to determine drug-mucin interactions using Caco-2, HT29, 2/4/A1, and Raji B cell lines. Unfortunately, the hierarchical structure of the mucosa was difficult to replicate in cell cultures (Liu et al. 2021; Sardelli et al. 2019). On the other hand, mono-component and poly-component artificial mucus models provide versatility and repeatability, despite their perceived current shortcomings in mimicking mucus physiological behavior. This can be attributed to the abundance of commercial mucin and other components needed to simulate the chemical and physiological environment of native mucus. One major advantage of the mono-component derived mucus model over the physiological model is the ability to study the potential effect of mucus components on the overall transport properties and to determine drug interaction with mucus components (Sardelli et al. 2019).

Under physiological conditions, the mucus operates as a molecular sieve in a network of mucins against foreign particles. Tangled and cross-linked mucins form a network to produce selective molecular sieves with potential binding sites. As a result, the movement of drug molecules through these mucin networks depends on both their size and surface chemical properties. Most molecules with size smaller than albumin (MW: 65 KDa; diameter 6–8 nm) can freely diffuse through the mucus layer because their permeation is not restricted by the molecular sieve properties of the mucin network. All peptides (MW = 500–1400 Da) investigated here are highly unlikely to be trapped within the mucin molecular sieve. Therefore, it is necessary to consider the drug interaction with protein-rich and glycans regions as modulators of drug diffusion (Cone 2009; Kishimoto et al. 2022; Olmsted et al. 2001; Desai et al. 1992).

It has been suggested that mucin interacts differently with colloidal particles containing opposite charges. The movement of positively charged nanoparticles across mucus was hindered because of their interaction with the negatively charged mucin. In contrast, negatively charged nanoparticles move more freely in both physiological and mono-component artificial mucus models (Crater and Carrier 2010). In this study, the negatively charged FITC-ADT10 peptide has higher diffusion than the positively charged FITC-HAV10 through the mucin (Fig. 3C). This is consistent with FITC-HAV10 likely forming an electrostatic interaction with the negatively charged glycan within the PTS domain of mucin. This is consistent with the observation of positively charged colloidal nanoparticles. This also might explain the intestinal mucosa permeation delay in T_max_ of FD4 when co-administered with HAVN1 peptide in our previous study (Dening et al. 2021). HAVN1 peptide is a cyclic peptide derivative of FITC-HAV10 peptide, and it also has slow permeation through the mucus layer to reach the intestinal epithelial barrier for opening its intercellular junctions to allow the penetration of FD4 across the paracellular pathway of the epithelial cell barrier of the intestine (Dening et al. 2021).

Mucin also influences the bioavailability of lipophilic cyclic peptide drugs (e.g., Cyclosporin A or CsA) because mucin hinders diffusion of CsA through the mucus barrier to reach the underlying epithelial cells of the intestine (Kishimoto et al. 2022). CsA can aggregate with mucins at the hydrophobic protein-rich region (Kishimoto et al. 2022). In this study, a similar behavior was observed with octreotide, octreotide A5, lanreotide, and lanreotide A5 (Fig. 4). The charge and cLogP of octreotide and octreotide A5 could influence their diffusion across the mucin layer. Octreotide has a predicted cLogP of 1.69 and a measured cLogP of 1.0, while the predicted cLogP of octreotide A5 is 1.99, both moderately hydrophobic. Interestingly, the permeation of octreotide A5 through the mucin layer was higher than octreotide; thus, the lipophilicity of both peptides is probably playing a minor role in the permeation (Fig. 4C). A similar observation was found with lanreotide and lanreotide A. Lanreotide has calculated and measured LogP of 3.35 and 2.5, respectively, while the calculated lanreotide A is 3.65. Comparing the calculated cLogPs of lanreotide and lanreotide A suggests lanreotide A is more lipophilic; however, lanreotide A diffuses more readily through mucin compared to lanreotide (Fig. 4F). On the contrary, mutation of Lys5 positive charge with the Ala residue in octreotide A5 improved its penetration through mucin. Therefore, the overall result suggests that the positive charge in octreotide and lanreotide hinders their penetration across mucin. The proposed explanation is also supported by the higher permeation of the negatively charged FITC-ADT10 than the positively charged FITC-HAV10 (Fig. 3C). Overall, the interaction between the positive charge in these peptides and the negative charge in mucin retards their permeation and the hydrophobicity of these peptides has less influence on their mucin permeability.

This study has found that the rank diffusion of ADT peptides has the following order: DTP-PVK > DTPPD > DTPPT > ADTC5. The results can be explained using the hydrophilicity and charge of each peptide. The calculated cLogPs of ADT peptides show the following order: DTPPVK = −1.49, DTPPT = −0.63, DTPPD = −0.50, and ADTC5 = −0.20. The neutral and high hydrophilicity DTPPVK peptide diffused through the mucus layer more readily than negatively charged and lower hydrophilicity peptides such as DTPPD, DTPPT, and ADTC5 peptides. The permeation of the hydrophilic peptide is usually less affected by the mucin compared to the more lipophilic peptides, which is in agreement with other reports (Falavigna et al. 2020; Khanvilkar et al. 2001). Unlike hydrophobic molecules, which form multiple low-affinity adhesive interactions with the hydrophobic region along mucin, hydrophilic molecules do not form strong additional interactions, because the hydrated glycan shell does not favor further hydrophilic interactions (Lai et al. 2009). The DTPPT and DTPPD peptides have similar hydrophilicity according to the calculated cLogP values; however, the DTPPD peptide with two negative charges has better diffusion than a single negatively charged DTPPT peptide. This observation can be explained due to the repulsive nature between two negative charges of DTPPD and the negative charges of mucin compared to that of a single charge of DTPPT. The higher hydrophilicity of DTPPT (cLogP = −0.63) compared to ADTC5 (cLogP = −0.20) could explain why DTPPT has better permeation through mucin than ADTC5 (Fig. 4E).

We proposed that positively charged lipophilic peptides interact the most with mucin, and this can be attributed to the dual effect of lipophilicity and positive charge interaction with the negatively charged mucin glycans. In addition, the hydrophilicity and charge repulsion can also explain the permeation of peptides through the mucin layer. Overall, the model successfully differentiated peptide diffusion based on physicochemical characteristics and mucin interactions. It was also shown that all three new cyclic ADT peptides exhibited fewer interactions with mucin compared to ADTC5, suggesting that these peptides may be more favorable peptides for modulation of intercellular junctions of the intestinal mucosa for enhancing oral drug delivery.

## Conclusion

In this study, we developed a simple and straightforward mucin model that was used to investigate peptide-mucin interactions. This model can differentiate permeation of peptides with different hydrophilicity and charge. This is a basic model that can be modified later by adding additional mucus components to better understand the role of each component. The model can be used to understand which physicochemical characteristics of peptides are favorable for a peptide’s mucus penetration for oral delivery. This study found that lipophilic and positively charged peptides interact the most with mucin and are likely to experience retarded transport through mucus. In contrast, neutral hydrophilic or negatively charged hydrophilic peptides exhibited the lowest interaction with mucin, and they are likely to pass easily through the mucus layer. Further studies are needed to investigate the effect of peptide solubilities and biorelevant media on these interactions will be investigated as well.

## Figures and Tables

**Table 1:** Peptide Names and Sequences

Peptide	Sequence	Molecular Weight (Da)	Net Charge at pH 6.8
FITC-HAV10	FITC-LFSHAVSSNG-NH_2_	1409	**+ 0.1**
FITC-ADT10	FITC-QGADTPPVGV-NH_2_	1328	**− 1.0**
ADTC5	Cyclo(1,7)Ac-CDTPPVC-NH_2_	772	**− 1.0**
DTPPD	Cyclo(1,5)DTPPD	525	**− 2.0**
DTPPT	Cyclo(1,5)DTPPT	511	**− 1.0**
DTPPVK	Cyclo(1,6)DTPPVK	637	**0.0**
Octreotide	FCFWKTCT	1019	**+ 2.0**
Octreotide A5	FCFWATCT	962	**+ 1.0**
Lanreotide	XCYWKVCT*	1096	**+ 2.0**
Lanreotide A5	XCYWAVCT*	1039	**+ 1.0**

* X represents Naphthalene

## Data Availability

The datasets used and/or analyzed during the current study are available from the corresponding author upon reasonable request.
